# Pre- and postsynaptic signatures in the prelimbic cortex associated with “alcohol use disorder” in the rat

**DOI:** 10.1038/s41386-024-01887-2

**Published:** 2024-05-16

**Authors:** Ana Domi, Davide Cadeddu, Erika Lucente, Francesco Gobbo, Christian Edvardsson, Michele Petrella, Elisabet Jerlhag, Mia Ericson, Bo Söderpalm, Louise Adermark

**Affiliations:** 1 Institute of Neuroscience and Physiology, Department of Pharmacology, The Sahlgrenska Academy, University of Gothenburg, Gothenburg, 413 90 Sweden; 2 Addiction Biology Unit, Department of Psychiatry and Neurochemistry, Institute of Neuroscience and Physiology, The Sahlgrenska Academy, University of Gothenburg, Gothenburg, 413 45 Sweden; 3 Centre for Discovery Brain Sciences, University of Edinburgh, Edinburgh, EH8 9JZ UK; 4 Linköping University, Department of Biomedical and Clinical Sciences, Center for Social and Affective Neuroscience. Linköping University, Faculty of Medicine and Health Sciences, Linköping, Sweden; 5 The Clinic for Addiction and Dependency, Sahlgrenska University Hospital, Gothenburg, Sweden

**Keywords:** Reward, Neurotransmitters, Addiction

## Abstract

The transition to alcohol use disorder (AUD) involves persistent neuroadaptations in executive control functions primarily regulated by the medial prefrontal cortex. However, the neurophysiological correlates to behavioral manifestations of AUD are not fully defined. The association between cortical neuroadaptations and behavioral manifestations of addiction was studied using a multi-symptomatic operant model based on the DSM-5 diagnostic criteria for AUD. This model aimed to characterize an AUD-vulnerable and AUD-resistant subpopulation of outbred male Wistar rats and was combined with electrophysiological measurements in the prelimbic cortex (PL). Mirroring clinical observations, rats exhibited individual variability in their vulnerability to develop AUD-like behavior, including motivation to seek for alcohol (crit 1), increased effort to obtain the substance (crit 2), and continued drinking despite negative consequences (crit 3). Only a small subset of rats met all the aforementioned AUD criteria (3 crit, AUD-vulnerable), while a larger fraction was considered AUD-resilient (0 crit). The development of AUD-like behavior was characterized by disruptions in glutamatergic synaptic activity, involving decreased frequency of spontaneous excitatory postsynaptic currents (sEPSCs) and heightened intrinsic excitability in layers 2/3 PL pyramidal neurons. These alterations were concomitant with a significant impairment in the ability of mGlu2/3 receptors to negatively regulate glutamate release in the PL but not in downstream regions like the basolateral amygdala or nucleus accumbens core. In conclusion alterations in PL synaptic activity were strongly associated with individual addiction scores, indicating their role as potential markers of the behavioral manifestations linked to AUD psychopathology.

## Introduction

Alcohol use disorder (AUD) is a complex psychiatric condition involving harmful alcohol use transitioning to a chronic relapsing profile even after extended periods of abstinence [1]. Long-term neuroadaptations in the medial prefrontal cortex (mPFC) have repeatedly been implicated in the manifestation of the transition into compulsive drinking [2–6]. The mPFC represents the primary neural site for value-based decision making and impulse control by integrating excitatory inputs from multiple subcortical regions [7]. Anatomical and functional studies in AUD patients reveal alterations in PFC neuronal structure and connectivity that may explain a lack of executive control over compulsive urge to consume alcohol [3, 8–10].

Research conducted on animals has shown that changes in mPFC excitatory glutamate signaling promotes the shift from controlled alcohol consumption to compulsive drinking [5, 11–13]. In heavy drinkers both increases and decreases in extracellular glutamate levels in the mPFC have been observed and recognized as critical components of AUD pathophysiology [14–16]. Especially, excessive alcohol consumption impairs synaptic transmission and neuronal excitability of glutamatergic pyramidal neurons, which constitute the majority of neuronal populations ( >70%) in the mPFC [17–25]. These changes in excitatory neurotransmission result from a complex interplay of different molecular mechanisms involving pre- and postsynaptic neuronal events and astrocytic activity [26]. However, most of these findings derive from studies of rodents passively exposed to alcohol or undergoing short periods of self-administration, whereas assessment of multiple signs of loss of control over alcohol intake most likely requires prolonged voluntary drinking that mimics human condition [27, 28].

The clinical manifestations for AUD are described in the Diagnostic and Statistical Manual of Mental Disorders (DSM-5) and encompass key features as: excessive alcohol drinking despite negative consequences, and alcohol craving [29]. We recently integrated these clinical signs into a multisymptomatic DSM-5-based rodent model monitoring 1) inability to abstain from alcohol-seeking, 2) motivation for alcohol and 3) persistent use despite punishment by electric foot-shock [30, 31]. We observed substantial behavioral differences in rat sub-populations possessing vulnerability and resilience toward alcohol addiction-like behavior. However, the underlying neuronal mechanisms of AUD vulnerability or resilience are not yet clear.

In the present study, we aimed to define neurophysiological correlates associated with an individual’s risk of manifesting AUD-like behavior in adult rats. We focused on the prelimbic (PL) mPFC subregion which is thought to play a role in the manifestation of drug seeking behaviors [2, 32]. We combined whole-cell and field potential measurements in the PL of rats categorized for their resilience or vulnerability to AUD-like behavior. Finally, we tested mGluR2/3 function by performing ex vivo pharmacological manipulations in PL and its downstream regions such as nucleus accumbens core (NacC) and basolateral amygdala (BLA). We hypothesized that unique changes in the PL synaptic profile would associate with AUD susceptibility.

## Methods and Materials

### Experimental procedures

**Fig. 1:**
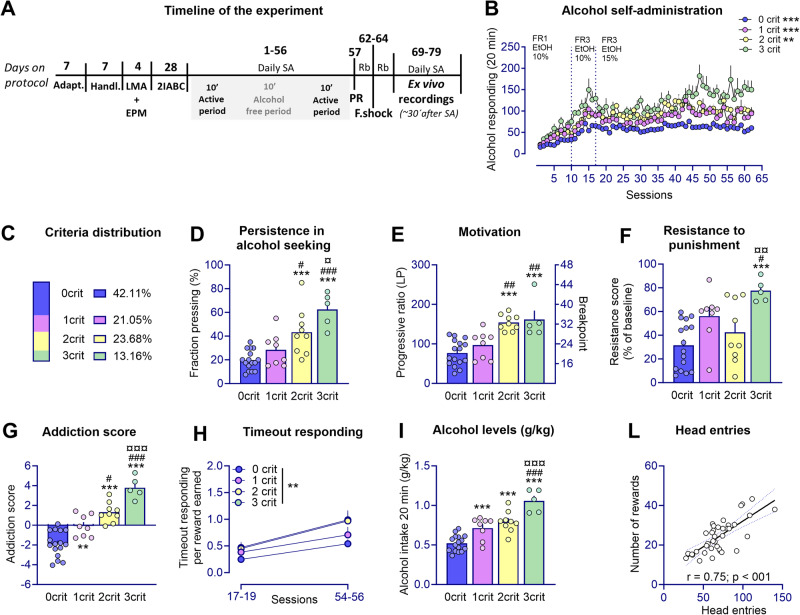
Addiction-like phenotypic traits emerge in a small subset of outbred rats after a long history of alcohol Intake. **A** Timeline for the experimental procedures that include behavioral and neurophysiological assessments (*Abbreviations:* Adapt Adaptation, Handl Handling, LMA Locomotor activity, EPM Elevated plus maze, 2IABC Intermittent access in a two-bottle choice procedure, SA Self-administration, PR Progressive ratio, F. shock Foot shock, Rb Rebaseline). **B** Rats (*n* = 38) acquired and maintained stable alcohol self-administration levels under a FR-1 (day 1–9) and fixed ratio-3 (FR-3; day 9–62) schedule of reinforcement. Active lever responding was significantly higher in 3crit rats as compared to 0crit,1crit (***p < 0.001), and 2crit rats (**p < 0.01). **C** Total distribution of animals depending on the criteria met (0–3crit): the largest proportion of rats fell into the 0crit group (42,11%) followed by the 1crit group (21.05%), the 2crit group (23.68%) and a small subset that met all the three criteria 3crit (13,16%). **D**–**F** There were significant differences in behavioral performance for each criterion and (**G**) the addiction score, particularly between rats that reached none of the criteria (resistant phenotype; 0crit) and animals that reached all three criteria (vulnerable phenotype; 3crit), (**p < 0.01, ***p < 0.001, significant as compared to 0crit vs 1,2,3 crit rats. ^#^*p* < 0.05, ^##^*p* < 0.01, ^###^*p* < 0.001 significant as compared to 1crit vs 0,2,3 crit rats. ^¤^*p* < 0.05, ^¤¤^*p* < 0.01, ^¤¤¤^*p* < 0.001 significant as compared to 2 crit vs 0,1,3 crit rats). **H** Timeout responding per reward earned differed significantly between groups and over time (**p < 0.01, group effect). **I** Daily alcohol intake (g/kg) was significantly different between criteria groups (0crit vs 1,2,3 crit rats, ***p < 0.001; 1crit vs 0,2,3 crit rats, ^###^*p* < 0.001; 2crit vs 0,1,3crit rats, ^¤¤¤^*p* < 0.001) and (**L**) the number of head entries and alcohol rewards correlated significantly.

Experimental procedures are schematically summarized in Fig. 1A and detailed in supplementary methods. Before alcohol exposure, anxiety-like behavior and locomotor activity in a novel environment were assessed to identify potential predictors of the AUD-like phenotypic trait. This was followed by a prolonged alcohol self-administration period at the end of which rats were screened for their addiction-like behavior according to the three DSM-5 AUD-like criteria. Following the final self-administration session, whole-cell patch-clamp and field potential electrophysiological recordings were conducted in the rats´ prelimbic cortex to explore the neurophysiological underpinnings of AUD-like behavior. In an additional experiment we examined PL activity in rats subjected to three footshock punishment sessions to mitigate potential bias from the footshock sessions when examining neurophysiological adaptations.

### Subjects

A total of 60 (40 SA, 20 controls) male Wistar rats (230–250 g) were housed in pairs under a 12 h reversed light/dark cycle. Food and water were provided *ad libitum*. Behavioral experiments were conducted during the dark phase (ZT12 to ZT15) of the light/dark cycle. Procedures followed the Swedish National Committee for Animal Research guidelines and approved by Local Ethics Committee for Animal Care and Use at Gothenburg University.

### Behavioral procedures

#### Experiment 1. Assessment of behavioral/personality traits prior to alcohol exposure

Locomotor activity (LMA) in a novel environment was assessed in a single 60 min session using the open field apparatus and considering total distance travelled in the first 30 min.

Anxiety-like behavior was evaluated in a five-minute test on the elevated plus maze (EPM), measuring time spent in open arms and number of open arm entries.

#### Experiment 2. Alcohol training and evaluation of the three criteria for AUD-like behavior

Rats (*n* = 40) were trained to self-administer ethanol (EtOH 10% on a fixed-ratio 1 (FR1) schedule of reinforcement followed by FR3 and EtOH 15% to sustain higher levels of alcohol responses [33, 34]). Training sessions lasted 30 min, consisted of two 10 min drug-period separated by a 10-min no-drug-period [31]. Pressing the active lever during the drug period resulted in 0.1 ml EtOH delivery, activation of a cue light above the lever for 5 s and a 10 s timeout period. Presses on the active lever during this 10‐second timeout period had no consequences and were recorded as timeout responding. “Inactive” lever responses were recorded but had no consequence. During the 10-min no-drug period, signaled by house light activation, pressing the active lever had no scheduled consequence.

*The evaluation of the three criteria for AUD-like behavior was the following:*

i. **Persistence of response** was evaluated daily by measuring active lever responding during the signaled no-drug 10-min periods and reported as “fraction pressing”. During the no-drug 10-min periods the house light was on and pressing the active and/or inactive lever led to no consequences. For each subject the daily value of lever pressing was classified as either “pressing” = 1 or “non-pressing” = 0, based on whether it exceeded the 66th percentile of the distribution among the entire population for that day averaged over a span of five days to mitigate fluctuations. We computed the “fraction pressing” as the algebraic sum of the “pressing” days expressed in percentage with respect to the total number of days for each individual rat.
ii. **Motivation** for alcohol was measured in a single progressive ratio (PR) schedule of reinforcement session, in which the response requirement to receive one dose of alcohol was increased as follows: for each of the first four alcohol deliveries, the ratio was increased by one; for the next four deliveries the ratio was increased by two; for all the following deliveries the ratio was increased by four [31].
iii. **Resistance to punishment** was measured in three consecutive 10 min sessions and we calculated the resistance score (Rs) by comparing average reward numbers earned in the initial 10 min across three baseline sessions and three punished schedule sessions [31]. Here, in a fixed ratio 3 (FR3) schedule of reinforcement, the first active lever press led to the illumination of a new, different green cue light stimulus signaling the presence of the shock session. The second active lever press produced a foot-shock of (0.25 mA, 0.5 s). The third active lever press produced the delivery of alcohol paired with a 5 s illumination of the cue light above the lever. If animals did not complete an FR3 within one minute, the green light turned off and the sequence was reinitiated.

A rat was considered positive for a specific addiction-like criterion when its behavior score ranked within the top 34% percentile of the distribution. This criterion was arbitrarily chosen based on seminal work from Deroche-Gamonet et al. (2004) [30], and considering that an arbitrary change of the selection threshold from 25 to 40% has minimal effect on individual rat-group allocation [35]. As a second level of analysis, we measured the addiction score by calculating the sum of the normalized score (z-score) of each criterion for each subject [36].

### Ex vivo electrophysiology

Following the evaluation of rats’ resistance to punishment, which was the last addiction-like criteria, rats received at least four additional SA sessions and were then taken for electrophysiological recordings, approximately 30 min after their last SA session. Water drinking rats, housed in parallel, were used as controls (ctrl). Ex vivo electrophysiology recordings were conducted in 250 μm thick coronal brain slices containing layer 2/3 of the PL. Brain slices containing NacC and BLA were used as controls to evaluate the effects of LY-354740 in downstream regions.

#### Whole-cell recordings

Layer 2/3 PL pyramidal neurons were visualized under infrared-differential interference contrast with a Nikon Eclipse FN1 microscope. Recording pipettes (resistance 2.5 to 5.5 MΩ) were filled with internal solution containing (in mM): 135 K-Glu, 20 KCl, 2 MgCl2, 0.1 EGTA, 10 Hepes, 2 Mg-ATP and 0.3 Na-GTP, with pH adjusted to 7.3 with KOH, and osmolarity to 295 mOsm with sucrose [37]. To record spontaneous excitatory postsynaptic currents (sEPSCs) in voltage clamp mode, neurons were clamped at −65 mV using MultiClamp 700B amplifier, digitized at 10 kHz and filtered at 2 kHz using Clampex. In a subpopulation of neurons, a current clamp protocol followed the voltage clamp recordings. Current was injected with a duration of 1000 ms and increasing intensity (intervals of 20 pA) from −80 to 240 pA to hyperpolarize and depolarize the neuronal membrane.

#### Field potential recordings

In field potential recordings population spikes (PS) were evoked with a stimulation frequency of 0.05 Hz in PL, NacC and BLA [38]. Stimulation electrodes were positioned locally, 0.2–0.3 mm from the recording electrode (resistance 2.5 to 4.5 MΩ), and the amplitude of evoked PSs were measured. To assess changes in mGluR2/3 signaling, slices were perfused with the mGluR2/3 agonist LY-354740 hydrate (100 nM). To monitor changes in inhibitory tone, slices were treated with the GABA_A_ receptor antagonist bicuculline-methiodide (bicuculline) (20 μM). When assessing responsiveness to LY-354740 and bicuculline, the stimulus intensity was set to yield a PS amplitude approximately half the maximal evoked response. A stable baseline was observed for 10 min before the 25 min drug perfusion. To assess changes in release probability after drug treatment, we used a paired pulse stimulation protocol (0.1 Hz, 50 ms interpulse interval).

### Statistics

See supplementary methods.

## Results

### AUD-like phenotypic traits emerge in a small subset of outbred rats after a long history of alcohol intake

After prolonged training ( >60 SA sessions) (Figs. 1A, B, S1) rats were scored for the three criteria that model the clinical signs of loss of control over alcohol drinking. We obtained four distinct groups of rats based on the number of criteria fulfilled (from 0crit to 3crit). The largest proportion of rats fell into the 0crit group (42.11%) followed by the 1crit group (21.05%) and the 2crit group (23.68%). Only a small percentage of animals met all three criteria (3crit;13.16%) (Fig. 1C). The 0crit and 3crit rats represented the two opposite extremes in each of AUD-like criteria tested. Persistence in alcohol seeking was significant different between groups (F_(3,34)_ = 16.64, *p* < 0.001; Fig. 1D, S2). “Fraction Pressing” rates were low for 0crit (19.69 ± 2.09) and progressively increased across 1crit (28.44 ± 4.99), 2crit (43.33 ± 6.60), and 3crit (62.50 ± 6.52) groups, with 3crit rats exhibiting higher non-reinforced responding (*p* < 0.001 vs 0crit and 1crit, *p* < 0.05 vs 2crit). In PR contingency, motivation for alcohol differed between groups (F_(3,34)_ = 14.75, *p* < 0.001; Fig. 1E) with higher levels of active responding observed in the 3crit rats (161.6 ± 22.84) compared to 0crit (77.06 ± 8.11, *p* < 0.001) and 1crit (97.13 ± 12.40, *p* < 0.001) rats but not differing from 2crit rats (154.13 ± 6.63, *p* = ns). In resistance to punishment resistance scores (Rs) were significantly different between groups (F_(3,34)_ = 6.91; *p* < 0.001, Fig. 1F). Punishing of operant responding decreased motivation for alcohol more in the 0crit (Rs: 31.57 ± 4.97, *p* < 0.001) 1crit (Rs: 56.12 ± 7.67, *p* < 0.05) and 2crit group (Rs: 42.57 ± 8.77, *p* < 0.01) as compared to 3crit group (Rs: 77.59 ± 4.24) Fig. 1F). Interestingly, while the other groups showed reduced responding over time, 3crit rats exhibited unwavering persistence in lever pressing throughout the three punished sessions (Fig. S3).

Based on the sum of normalized scores (z-scores) assigned to each criterion we obtained an addiction score for individual rats (Fig. 1G) [31, 36]. The addiction score was negative for 0–1crit groups (0crit = −1.88 ± 0.32 and 1crit = −0.07 ± 0.39) and positive for 2-3crit groups (2crit = 1.31 ± 0.33 and 3crit = 3.79 ± 0.49) (F_(3,34)_ = 34.85, *p* < 0.001; 0crit vs 1crit, *p* < 0.01; 0crit vs 2crit and 3crit, *p* < 0.001; 1crit vs 2crit, *p* < 0.05 and 1crit vs 3crit, *p* < 0.001; 2crit vs 3crit, *p* < 0.001). A high correlation between addiction score and distribution of animals within the criteria indicates the interdependence between these two measures (r = 0.8635, *p* < 0.001).

Daily active lever presses during 10-s timeout periods were used to examine the inability to withhold inappropriate responses [39, 40]. Generalized linear mixed model (GLMM) analysis on timeout responding lever presses normalized by the number of rewards showed that whilst controlling for rewards earned there is as significant effect of group (F_(3, 68)_ = 5.66, *p* < 0.01) and time (F_(1, 68)_ = 26.32, *p* < 0.001) with respect to inability to withhold responses. Post hoc analysis showed a significant difference in timeout responding between the 0crit and 2crit group (df=68, t = 3.078, *p* = 0.018), and a similar trend was observed between 0crit and 3crit (df=68, t = 2.457, *p* = 0.079). Additionally, there was a significant effect of time across all groups (df=68, 0crit: t = 3.533 *p* = 0.001, 1crit: t = 2.038 *p* = 0.045, 2crit: t = 2.625 *p* = 0.011) except for the 3crit rats that nevertheless approached significance (t = 1.904 *p* = 0.061) (total active lever presses presented in Fig. S4).

Finally, we evaluated the relationship between alcohol intake (g/kg) and propensity to develop addiction-like behavior. We found a main effect for the amount of alcohol consumed with respect to criteria subgroup (F_(3,34)_ = 27, *p* < 0.001), with 3crit rats consuming more compared to the other groups (*p* < 0.001) (Fig. 1I). The number of head entries further correlated with the number of alcohol deliveries (average last three days) (r = 0.75, *p* < 0.001) (Fig. 1L).

### Proximity of rats’ AUD-like behavioral profile in a principal component analysis

**Fig. 2:**
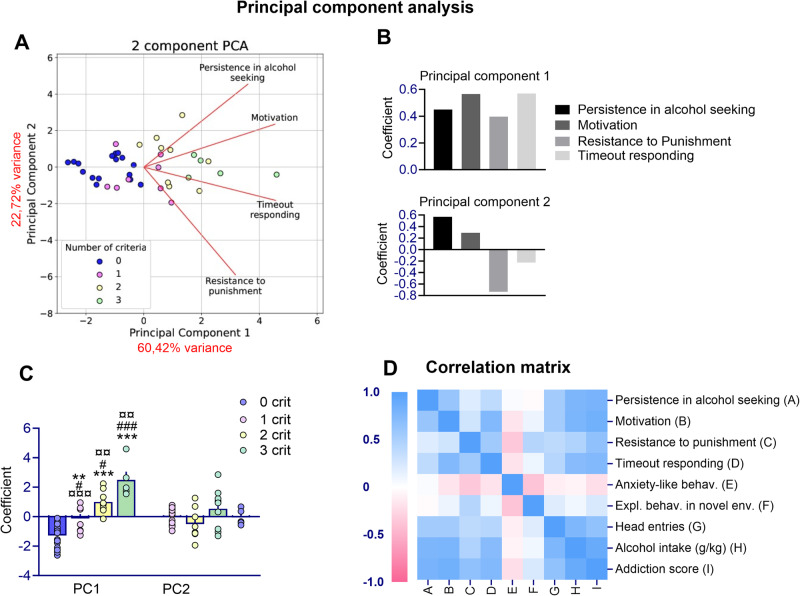
Proximity of rats’ addiction-like behavioral profile in a Principal Component Analysis and correlation analysis. **A** The three addiction-like criteria and timeout responding clustered according to the loading in two components of the PCA with (**B**) their respective coefficients. PC1 accounted for more than 60% of the total variance with the behavioral measures similarly represented in this component, while PC2 accounted for only 23% of the variance, with resistance to punishment being the main criterion represented. **C** PC1 was able to significantly discriminate the animals depending on the criteria met while PC2 did not (**p < 0.01, ***p < 0.001, significant as compared to 0crit vs 1,2,3 crit rats, ^#^*p* < 0.05, ^###^*p* < 0.001 significant as compared to 1crit vs 0,2,3 crit rats and ^¤¤^*p* < 0.01, ^¤¤¤^*p* < 0.001 significant as compared to 2 crit vs 0,1,3 crit rats). **D** Correlation matrix of various behavioral measures.

Principal Component Analysis (PCA) was performed to examine the interdependence between behavioral measures in animals, focusing on addiction-like criteria and timeout responding. The first two components explained 83.15% of the total variance, with PC1 contributing over 60%, making it the most representative factor (Fig. S5). The factor loadings for persistence in alcohol seeking (45%), motivation (56%), resistance to punishment (40%) and timeout responding (57%) were similarly represented in this component, indicating that it reflects the addiction-like behavior construct. This finding aligns with previous analyses from studies employing the 0/3 crit addiction model, where the addiction-like criteria were inter-related and loaded onto a single factor [30, 41]. PC2 counted only for 22.72% of the total variance with resistance to punishment being the main criterion represented (73.6%) (Fig. 2B). PC1 was able to discriminate subjects depending on the criteria met (F_(3,34)_ = 29.82, *p* < 0.001), with significant different coefficient loading between each criteria group (0crit vs 1crit, *p* < 0.01; 0crit vs 2crit and 3crit, *p* < 0.001; 1crit vs 2crit, p < 0.05 and 1crit vs 3crit, *p* < 0.001; 2crit vs 3crit, *p* < 0.01).While there were no differences in coefficient loading between the criteria groups in PC2 (F_(3,34)_ = 1.75, *p* = ns) (Fig. 2C).

### Anxiety-like response and exploratory behavior are predictor traits for alcohol seeking despite punishment

We retrospectively examined predictive relationships between vulnerability traits assessed prior to alcohol exposure and subsequent development of AUD-like behavior. Alcohol seeking despite punishment was the only criteria showing significant positive correlation with novelty-induced LMA (r = 0.50, *p* < 0.001) (Fig. 2D). Similar to humans, rats exhibiting high levels of “sensation seeking” demonstrated increased alcohol seeking behavior despite negative consequences [42]. Moreover, we found a negative correlation between percentage of time spent in the EPM open arms and resistance to punishment (r = −0.35, *p* < 0.05) (Fig. 2D).

### Selective disruption of glutamatergic synaptic activity in the prelimbic cortex of an AUD-prone phenotype following chronic alcohol intake

**Fig. 3:**
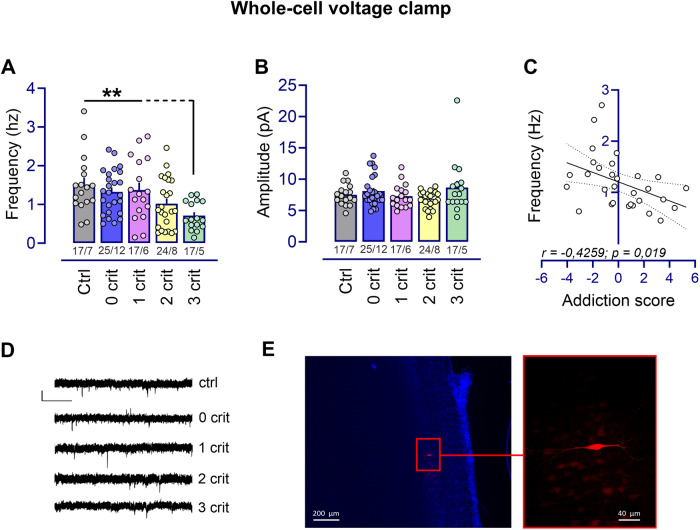
Spontaneous excitatory neurotransmission of PL layer 2/3 pyramidal neurons is altered exclusively in rats manifesting AUD-like traits. **A** Whole cell recordings performed in voltage-clamp mode showed that the frequency of spontaneous events was significantly reduced in the 3crit rats compared to the ctrl, 0crit and 1crit rats (**p < 0.01 significant as compared to 3crit vs ctrl, 0crit and 1crit rats) while (**B**) the amplitude of spontaneous events was similar across groups (*n* = x/y; x: number of recordings/y: number of rats recorded). **C** The sEPSC frequency significantly correlated with the addiction score of the recorded rats. **D** Representative traces showing measured sEPSCs for each recorded group. Calibration: 1 s, 10 pA. **E** Photomicrographs of a biocytin-filled pyramidal neuron. Scale bar 200 μm and 40 μm.

Electrophysiological whole-cell recordings in voltage-clamp mode showed a significant effect on sEPSC firing frequency as a function of criteria met and compared to water drinking ctrl rats (F_(4,94)_ = 4.39, *p* < 0.01) (Fig. 3A). The frequency of spontaneous events was significantly reduced in 3crit rats compared to ctrl (*p* < 0.01), 0crit (*p* < 0.01), 1crit (*p* < 0.01) but not 2crit (*p* = 0.14) rats. The sEPSC frequency was indistinguishable between ctrl rats never exposed to alcohol and those subjected to chronic alcohol consumption from 0crit,1crit and 2crit groups (ctrl vs 0crit,1crit and 2crit, p=ns). The determined sEPSC frequency correlated with the individual addiction score (r = −0.43, *p* < 0.05) (Fig. 3C), indicating that PL presynaptic input is directly associated with the behavioral manifestation of addiction-like criteria. Importantly, punishment did not influence sEPSC firing frequency as assessed in a separate batch of animals (*n* = 12) (Fig. S9). Amplitude (F_(4,94)_ = 1.78, *p* = 0.14) decay time (F_(4,94)_ = 1.94, *p* = 0.11) and rise time (F_(4,94)_ = 0.92, *p* = 0.45) of spontaneous events were not significantly different between ctrl and 0-1-2-3crit rats (Fig. 3B, Table S1). Absolute values of synaptic transmission, membrane properties and neuronal excitability of PL glutamatergic neurons are outlined in the supplementary information (Table S1).

**Fig. 4:**
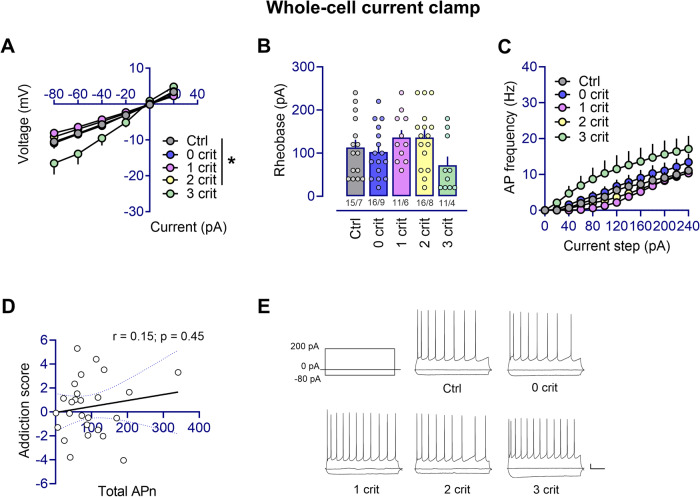
Neuronal excitability of PL layer 2/3 pyramidal neurons is increased exclusively in rats manifesting AUD-like traits. **A** Whole cell recordings performed in current-clamp mode showed that the relative change in membrane potential evoked by current injection was significantly bigger in pyramidal neurons from the 3crit rats as compared to the other groups (3crit vs ctrl, 0crit, 1crit, 2crit rats, *p < 0.05). **B** PL layer 2/3 pyramidal neurons also presented a trend toward reduced rheobase and (**C**) increased AP frequency in the 3crit rats compared to the other groups (n  =  x/y; x: number of recordings/y: number of rats recorded). **D** The total number of AP (APn) firing did not correlate with the addiction score of the recorded rats. **E** Representative traces of current clamp recordings from all groups. Calibration: 200 ms, 10 mV.

Current clamp recordings were performed to measure intrinsic excitability of PL-2/3 pyramidal neurons. Input resistance differed among groups (F_(4,64)_ = 3.63, *p* ≤ 0.01) with neurons from 3crit rats presenting higher input resistance compared to ctrl (*p* < 0.05), 0crit 1crit and 2crit (*p* < 0.01) rats (Table S1). Differences in membrane voltage were observed between groups (F_(4,64)_ = 4.33, *p* < 0.01; 0crit vs 2crit, *p* < 0.05; and 2crit vs 3crit, *p* < 0.05) while action potential (AP) threshold was not significantly affected (F_(4,64)_ = 0.63, *p* = 0.65) (Table S1). The I-V curve differed between groups (F_(4,64)_ = 3.48, *p* < 0.05) with 3crit rats showing a greater change in membrane voltage compared to the other groups (ctrl and 0crit vs 3crit, *p* < 0.05; 1crit and 2crit vs 3crit, *p* < 0.01) (Fig. 4A). This was accompanied by a trend toward decreased minimum current necessary to trigger an AP (rheobase: F_(4,63)_ = 1.99, *p* = 0.10) and a trend towards increased AP firing (F_(4,64)_ = 2.38, *p* = 0.06) (Fig. 4B, C). However, there was no correlation between the total number of action potential (APn) firing and the rats´ addiction score (r = 0.15, *p* = 0.45) (Fig. 4D).

### Neuroadaptations of mGluR2/3 and GABA_A_ receptor signaling following chronic alcohol intake: phenotype-dependent and phenotype-independent effects

**Fig. 5:**
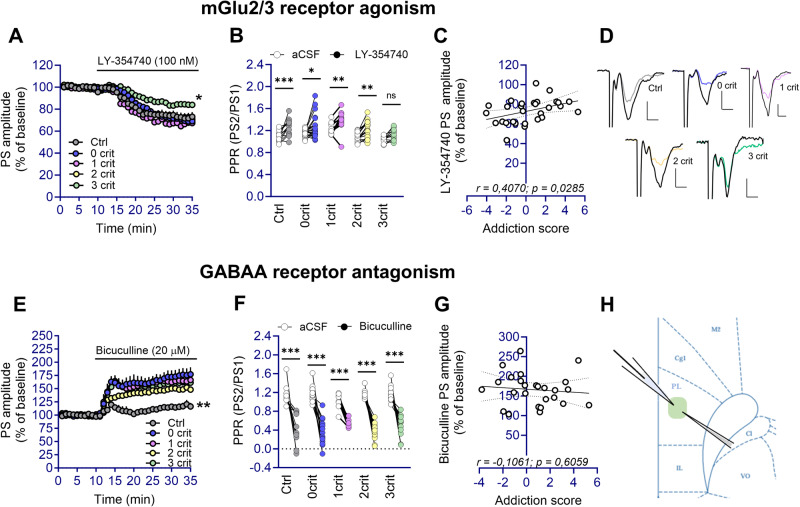
Synaptic depression induced by mGluR2/3 agonist is suppressed exclusively in rats manifesting AUD-like traits while GABA_A_R signaling adaptation is consistent across alcohol-exposed groups. **A** Synaptic depression induced by the mGluR2/3 agonist LY-354740 was significantly lower in the 3crit rats compared to the other groups (3crit vs ctrl, 0crit, 1crit, 2crit rats, *p < 0.05). **B** LY-354740 significantly increased PPR in all groups, apart from the 3crit group (*p < 0.05, **p < 0.01, ***p < 0.001). **C** Suppression of PS amplitude with LY-354740 correlated with the addiction score of the recorded rats. **D** Example traces based on a mean of 5 traces at baseline and after treatment with LY-354740 for each treatment group. Calibration: 2 ms, 0.2 mV. **E** Disinhibition induced by the GABA_A_R antagonist bicuculline, was significantly more pronounced in brain slices from rats exposed to alcohol compared to water drinking controls (ctrl vs 0crit, 1crit, 2crit and 3crit rats, **p < 0.01). **F** Bicuculline increased PPR in all groups (***p < 0.001). **G** PS amplitude of evoked potentials did not correlate with the addiction score of the recorded rats after bicuculline bath perfusion. **H** Schematic drawing depicting the area in the PL where recordings were performed.

A loss of mGluR2/3 function in the mPFC has been related to escalation of alcohol consumption in rodents, and alcoholism in humans [13, 43]. Activation of mGluR2/3 with LY-354740 induced phenotype-dependent synaptic depression of evoked potentials in the PL (group: F_(4,74)_ = 3.19, *p* < 0.05; time: F_(20,1480)_ = 122.08, *p* < 0.001; time x group: F_(20,1480)_ = 1.98, *p* < 0.001) (Fig. 5A), with a reduced response in brain slices from 3crit rats (3crit vs ctrl, 0crit and 2crit, *p* < 0.05; 3crit vs 1crit, *p* < 0.01). The blunted response was further confirmed by no change in release probability in 3crit rats in response to LY-354740 perfusion, but a significantly increased PPR in all other groups (aCSF vs LY-354740: ctrl, *p* < 0.005; 0crit, *p* < 0.05; 1crit and 2crit, *p* < 0.01; 3crit, *p* = 0.18) (Fig. 5B). In addition, the relative suppression of PS amplitude correlated with rats’ addiction scores (r = −0.4070, *p* < 0.05), thereby reinforcing the link between specific PL mGluR2/3 changes and AUD susceptibility (Fig. 5C). We further observed a correlation between mGluR2/3 agonist-induced synaptic depression and sEPSC frequency in voltage-clamp recordings, suggesting potential PL presynaptic adaptations through both these parameters (r = −0.53, *p* < 0.01) (Fig. S6).

Since chronic alcohol is known to affect neurotransmission in both the PL and its related projections, we also investigated possible neuroadaptations associated with mGluR2/3 signaling in NacC and BLA. Activation of mGluR2/3 in NacC and BLA showed comparable synaptic depression of evoked field potentials with no significant differences between groups (NacC: F_(4,51)_ = 0.72, *p* = 0.58; BLA:F_(4,70)_ = 0.74, *p* = 0.57) (Fig. S7). Given that all recordings were performed consistently on the same animals using daily identical procedures, these data suggest that any phenotype-dependent adaptations within the mGluR2/3 system are restricted to the PL and do not extend to downstream regions.

Lastly, possible changes in inhibitory tone were assessed in PL by bath perfusion of the GABA_A_ receptor antagonist bicuculline. Disinhibition of evoked potentials induced by bicuculline was higher in brain slices from all alcohol treated animals compared to water controls and was concomitant with a reduction in PPR in all groups (aCSF vs bicuculline: *p* < 0.001 for ctrl, 0-1-2-3crit) (Fig. 5E, F). Among alcohol criteria groups bicuculline disinhibited PL output to a similar extent suggesting that chronic alcohol exposure may disrupt GABA_A_R-dependent neurotransmission independently of an individual’s susceptibility to developing behaviors associated with AUD (Fig. 5G).

## Discussion

Alcohol use disorder stands out as one of the most demanding areas of unmet medical needs in psychiatry, primarily due to the elusive nature of its underlying neuronal mechanisms. The likelihood of developing AUD varies on an individual basis, and the data presented here demonstrate specific neuronal signatures correlating with behavioral manifestation of individual vulnerability to addiction-like behavior. Our results highlight important changes in PL glutamatergic synaptic activity and mGluR2/3 receptor signaling associated with the development of AUD-like behavior.

Parallel to human drug use patterns we found that AUD-like behavior emerged in only a fraction of subjects which replicated and strengthened prior findings from our research and that of others on the propensity to develop AUD-like behavior in rodents [31, 41, 44]. After prolonged alcohol self-administration, 13% of the population fulfilled the DSM-5 based addiction criteria involving persistence in alcohol-seeking when alcohol was not available, motivation for alcohol and continued drinking despite punishment. One limitation of our study is that we did not investigate whether 3crit rats exhibited habitual responding. Important additional experiments are needed in order to adapt the 0/3 crit alcohol model to study resistance to extinction and habit formation which are key process in the field of drug addiction [45].

Retrospective analysis of traits associated with AUD susceptibility demonstrated a correlation between resistance to punishment and elevated locomotor activity in a novel environment, coupled with increased time spent in the EPM closed arms. Hyperlocomotion in a novel environment may be linked to sensation-seeking traits and predicts psychostimulant but not opioids use [46]. In humans, heightened sensation seeking correlates with reduced sensitivity to negative outcomes, potentially explaining our link between response to novelty and resistance to punishment [47]. We further observed a negative correlation between percentage of time spent in the EPM open arms and resistance to punishment, aligning with prior studies on anxiety disorders and increased alcohol dependence risk [48]. However, to draw a conclusion when examining the relationship between anxiety-like behavior and prospective alcohol drinking, a broader array of anxiety tests beyond EPM observations is needed.

The propensity to develop AUD-like behavior related to the amount of alcohol consumed. Interestingly, these results were not only corroborated in our previous findings on outbred rats [31], but are also consistent with the work from Jadhav et al. (2017) [41] where Wistar rats’ addiction scores correlated with their alcohol lever presses. Similar findings on Lister Hooded outbred rats demonstrated that animals that consumed the highest levels of alcohol, exhibited higher resistance to punishment during quinine adulteration [49]. However, other studies, principally conducted in alcohol-preferring rats, do not link compulsive alcohol seeking to the drinking history [33, 34, 50]. One possible explanation is that alcohol-preferring rats, due to their heavy alcohol intake, might face a ‘ceiling effect’ making it challenging to detect individual consumption differences linked to the development of AUD-like behavior. Importantly, high alcohol intake is a major determinant of alcohol addiction in humans, and associated with fulfilling a greater number of DSM-IV/5 criteria [51, 52].

In the present study, 3crit AUD-like behavior was specifically associated with neurophysiological changes in the PL involving decreased frequency of sEPSCs and enhanced intrinsic excitability in layers 2/3 pyramidal neurons. This was coupled with an impairment in mGlu2/3 receptors’ ability to negatively regulate glutamate release. The changes in glutamatergic transmission were observed immediately after ( ~30 min) the rats´ last SA session thus they might not reflect later time points. However other studies have observed comparable PL synaptic changes immediately after alcohol consumption and after a few days of withdrawal [53, 54].

Recent research suggests that chronic alcohol exposure can induce distinct forms of neuronal plasticity when examining different layers in the rodents´ PL [22, 23, 55]. The results of our voltage-clamp recordings align with earlier observations from both male and female mice in which excessive alcohol drinking led to decreased sEPSC firing frequency in PL layer2/3 pyramidal neurons [56]. Furthermore, consistent with binge drinking studies we found no differences in sEPSC amplitude rise time and decay time between 0-3crit groups and water controls, indicating the absence of postsynaptic transformations [19, 56]. Changes in sEPSC frequency suggest possible alterations of presynaptic neurons from brain regions that project to PL [57, 58]. This might implicate a dampening of cognitive or behavioral processes that rely on this brain region increasing the risk of compulsive alcohol drinking. The reduction in sEPSC frequency correlated with individual addiction scores, suggesting a direct link between PL presynaptic activity and AUD-like behavior. Moreover, rats that self-administered alcohol over a long period but did not fulfil all AUD-criteria did not differ in sEPSC frequency from water drinking controls rats. This highlights the importance of a multicriteria approach in preclinical alcohol addiction research beyond just alcohol consummatory behavior to provide a more accurate representation of the human addiction neurobiological complexities. Importantly, a direct connection between reduced sEPSC frequency in the PL and AUD is further supported by studies of synaptotagmin 1 (SYT1), a membrane-trafficking protein playing a key role in transmitter release [59]. In fact, selective downregulation of SYT1 in PL, not only reduces the probability of transmitter release but also results in escalated alcohol consumption, increased motivation to consume alcohol, and increased drinking despite negative consequences [2].

In humans, group II metabotropic glutamate receptors mediate presynaptic inhibition of excitatory transmission in cortical pyramidal neurons [60]. Our findings reveal that activation of mGluR2/3 induced a weaker synaptic depression in the PL of the AUD-prone phenotype compared to the other groups, indicating a possible loss of function of these receptors. This is partially supported from analysis of post-mortem tissue from human alcoholics, showing reduced Grm2 expression, responsible for encoding metabotropic glutamate receptor2 (mGluR2) [13]. The association we identified between the amplitude of evoked potentials and individual addiction scores further supports the role of mGluR2/3 in the addiction development process. Dysfunctional mGluR2 in the medial prefrontal cortex is also linked to the emergence of cocaine addiction-like behavior, suggesting the involvement of this receptor system in broader behavioral manifestation of addiction [61]. We also demonstrated that impaired mGluR2/3 signaling in AUD-prone rats was localized to the PL and absent in downstream regions nucleus accumbens and basolateral amygdala where mGluR2/3 agonism-induced synaptic depression was similar among groups. Importantly, the relative depression induced by mGluR2/3 agonist correlated with sEPSC frequency. It is thus possible that the impaired signaling is associated with a floor effect, where sEPSC frequency is depressed to an extent where a further reduction is not possible. Another possibility could be that extrasynaptic glutamate levels are increased, resulting in activation of mGluR2/3, and thus the agonist may not further activate these receptors. Increased extrasynaptic glutamate could also contribute to the observed increase in excitability and is partially supported by studies monitoring cortical glutamate levels during withdrawal [62].

Long-term alcohol consumptions may also alter intrinsic neuronal excitability of PL neurons [19, 63] and the data presented here demonstrate increased input resistance specifically in the AUD-phenotypic trait accompanied by a trend in reduced cell capacitance and increased membrane resistance. In addition, 3crit rats displayed altered I/V relationships and showed a strong trend towards increased AP firing and reduced rheobase, indicating that the ability of PL pyramidal neurons to generate an AP was enhanced in rats displaying AUD-like behavior. An increase in neuronal excitability might be a factor driving alcohol compulsive seeking behavior [20, 64], although future studies are required to investigate the causal relationship. Intriguingly, neuroimaging studies in human alcoholics have revealed that alcohol-induced adaptations in the mPFC shift this region toward a hyperexcitable state, characterized by greater metabolic activity at rest [65]. Considering the regulation of excitatory neurotransmission by GABAergic interneurons, reduced spontaneous inhibitory transmission could partially underly the enhanced intrinsic excitability of the PL pyramidal neurons [20]. However, the GABA_A_ antagonist bicuculline disinhibited evoked potentials to a similar extent in all alcohol-exposed groups indicating that other intrinsic factors, such as changes in phosphorylation patterns might play a role [66]. This latter finding, i.e. the consistent disinhibition induced by the GABA_A_ antagonist across all alcohol-exposed groups, underscores the presence of phenotypic-independent alterations in synaptic signaling. This observation way reflects neuronal adaptations linked to prolonged alcohol exposure that are not directly related to the expression of AUD-like behavior. However, to draw a definitive conclusion, further investigation of the PL inhibitory transmission using the multisymptomatic AUD model is warranted.

In conclusion, using a multi-symptomatic model of addiction, we demonstrate that neuroadaptations in the prelimbic cortex occur exclusively in rats manifesting AUD-like traits.

To the best of our knowledge this is the first long-term voluntary alcohol consumption study in rats, correlating sub-dimensions of alcohol-like addictive behaviors, as defined by the DSM-5, with distinct adaptations in prelimbic glutamatergic neurotransmission. Our findings strive to bridge the translational gap in alcohol research holding great promise in guiding drug development efforts aimed at addressing the complexities of alcoholism among affected individuals.

## Supplementary information

Supplementary information
