# Supplementary material for: Pre- and postsynaptic signatures in the prelimbic cortex associated with “alcohol use disorder” in the rat
Source: Neuropsychopharmacology. 2024 May 16;49(12):1851–60. doi: 10.1038/s41386-024-01887-2 (PMC11473806; doi:10.1038/s41386-024-01887-2)
Supplement: Supplementary file 1 — Supplementary information [file 41386_2024_1887_MOESM1_ESM.pdf]

Pre- and postsynaptic signatures in the prelimbic cortex associated with “alcohol use disorder” in the rat

Authors:

Ana Domi[1,2] *, Davide Cadeddu[1,] Erika Lucente[1], Francesco Gobbo[3], Christian Edvarsson[1], Michele Petrella[4], Elisabet Jerlhag[1], Mia Ericson[1,2], Bo Söderpalm[2,5], Louise Adermark[1,2]

[1]. Institute of Neuroscience and Physiology, Department of Pharmacology, The Sahlgrenska Academy, University of Gothenburg, Gothenburg 413 90, Sweden

[2] Addiction Biology Unit, Department of Psychiatry and Neurochemistry, Institute of Neuroscience and Physiology, The Sahlgrenska Academy, University of Gothenburg, Gothenburg 413 45, Sweden

[3] Centre for Discovery Brain Sciences, University of Edinburgh, Edinburgh EH8 9JZ, United Kingdom

[4] Linköping University, Department of Biomedical and Clinical Sciences, Center for Social and Affective Neuroscience. Linköping University, Faculty of Medicine and Health Sciences.

[5] The Clinic for Addiction and Dependency, Sahlgrenska University Hospital, Gothenburg, Sweden.

Corresponding author:

PhD Ana Domi

Department of Pharmacology,
University of Gothenburg, Sahlgrenska Academy
Medicinaregatan 13, Sweden
Tel: +46 738977316
email: ana.domi@gu.se

SUPPLEMENTARY MATERIAL AND METHODS

Subjects

Sixty male Wistar rats (Charles River, Germany), weighing 230-250g at the start of the experiments, were used in this study. Rats were housed in pairs under a 12-hour (ZT12 to ZT24) reversed light/dark cycle (lights off at 8:00 AM). Food and water were provided ad libitum. Experiments were conducted during the dark phase of the cycle and all efforts were made to minimize rats' suffering and distress. At arrival, rats were left undisturbed to acclimatize at the animal facility for one week. The following week rats were handled twice a day prior to any behavioral procedure. Procedures were conducted in accordance with the National Committee for Animal Research in Sweden and approved by the Local Ethics Committee for Animal Care and Use at Gothenburg University (Dnr 5.8.18-13023/2019).

Drugs

The alcohol solution was prepared by mixing 95% ethanol (Kiilto Clean AB, Täby, Sweden) with tap water to obtain 10% and 15% alcohol. The mGluR2/3 agonist LY-354740 hydrate (Tocris, Bristol, UK) was dissolved in Milli-Q to 5 mM and further diluted in artificial CSF (aCSF) to final concentration (100 nM). The $GABA_A$ receptor antagonist bicuculline-methiodide (bicuculline) (Sigma Aldrich, Stockholm, Sweden) was diluted in Milli-Q to 20 mM and further diluted in aCSF (20 μM).

Behavioral procedures

Open field and elevated plus maze

The open field (OF) apparatus comprised sound-attenuated chambers housing a 40 × 40 cm arena equipped with infrared beam detectors (Med Assoc., Fairfax, VT, USA). Locomotor activity (LMA) was tracked and recorded using activity monitor 7 (Med Assoc., St. Albans, VT, USA). Rats were placed in a corner of the OF and allowed to freely explore for 60 minutes. Novelty-induced locomotor activity was measured by the total distance travelled during the first 30 minutes of OF exposure.

The elevated plus maze (EPM) was made of black plexiglass and consisted of two open arms and two enclosed arms (40 cm high walls) arranged so that the similar arms were opposite each other. The maze, elevated 50 cm above the floor, was located in a sound attenuated room illuminated by a red dim light. At the beginning of the test each rat was placed in the center platform facing an open arm. The test lasted for five minutes during which the number of open and closed-arm entries and the time spent in each arm was recorded. The percentage of time spent in open arms and the number of open arm entries (with entries defined as placement of all four paws into the respective arm) were used as an index of anxiety-like behavior [1].

Both OF and EPM testing occurred during the dark phase, with the rats having undergone a one-hour prior habituation to the test room.

Operant alcohol self-administration

Before initiating the operant response training, 40 rats underwent a four-week intermittent two-bottle choice alcohol consumption procedure in their homecage. During this procedure, the rats were presented with a choice between a 10% alcohol solution and water. This training protocol was adopted to avoid sucrose fading or water deprivation procedures and facilitate the acquisition of operant responding [1].

Eight additional rats from the same cohort served as water control subjects throughout the entire study.

Alcohol self-administration was performed in rat operant conditioning chambers (Med Associate St Albans, VT, USA) enclosed in sound-attenuating, ventilated, environmental cubicles. Each chamber was equipped with two retractable levers located in the front panel of the chamber with two stimulus lights placed above each lever in addition to a house light near the top of the chamber opposite the lever and a tone generator. Alcohol receptacle was equipped with a sensor to detect head entries. Levers were extended at the beginning of the self-administration sessions. Infusions occurred by means of syringe pumps (Med Associates, Inc., St. Albans, VT) connected to plastic tubing that was connected to the receptacle. The number of operant responses of both the active and inactive levers, as well as the number of reinforcers received were recorded. The operant chambers were controlled, and data collected with MED-PCr IV windows-compatible software (Med Associates Inc – USA).

Rats (n=40) were first trained to self-administer ethanol (EtOH) 10% on a fixed-ratio 1 (FR1) schedule of reinforcement until stable baseline was reached (session n. 1-8). Then, the FR was increased to FR3 and EtOH 15% was introduced (starting at session n.17) to sustain higher levels of alcohol responses [2,3]. Training sessions were 30 min in duration, during which a 10-min reward-available period (drug-period) was followed by a 10-min reward-unavailable period (no-drug-period) which was followed by a second 10-min drug-period. Pressing the active lever during the drug period resulted in the delivery of 0.1 ml of EtOH followed by the activation of a cue light above the lever for 5 s and a 10 s time-out period. During the no-drug period, signaled by activation of the house light, pressing the active lever had no consequences. Responses on the ‘‘inactive’’ lever were recorded during the entire session but did not result in any programmed consequences. During the 10-second time-out periods, both active and inactive responses were recorded, with active lever presses serving as an indicator of impulsive-like responses [4]. Two rats were excluded from the study as they failed to acquire EtOH SA.

Evaluation of the Three Criteria for AUD Like-Behavior

Persistence of response

During training with EtOH 15% (session 17-56) persistence of response was evaluated daily by measuring active lever responding during the signaled no-drug 10-min periods in the absence of ethanol. During the no-drug 10-min periods the house light was on and pressing the active and/or inactive lever led to no consequences. Both active and inactive responses were recorded, with active lever presses serving as an indicator of persistence of response. In the specific for each subject the daily value was classified as either "pressing" = 1 or "non-pressing" = 0, based on whether it exceeded the 66th percentile of the distribution among the entire population for that day averaged over a span of five days to mitigate fluctuations. We

computed the “fraction pressing” as the algebraic sum of the “pressing” days expressed in percentage respect the total days for each individual rat.

Motivation

Motivation for alcohol was measured in a progressive ratio (PR) schedule of reinforcement. Under the PR condition the response requirement (i.e. the number of lever responses or the ratio required to receive one dose of 15% ethanol) was increased as follows: for each of the first four ethanol deliveries the ratio was increased by 1; for the next four deliveries the ratio was increased by 2 and for all of the following deliveries the ratio was increased by 4 (1, 1, 1, 1, 2, 2, 2, 2, 4, 8, 12, 16, 20, 24, 28, 32, 36, 40, 44, 48, 52, 56, 60, 64, 68, 72, etc). Each alcohol delivery was paired with a 5 s illumination of the cue light. Sessions were terminated when 30 min had elapsed since the last reinforced response. The maximal number of responses that a rat produced to obtain one infusion was referred to as the break point [1].

Resistance to punishment

During resistance to punishment rats were placed for 10 minutes in the SA chambers for three consecutive days. Here in a fixed ratio 3 (FR3) schedule of reinforcement the first active lever press led to the illumination of a new, different green cue light stimulus signaling the presence of the shock session. The second active lever press produced a foot-shock of (0.25 mA, 0.5 s) via a metal grid connected to a shock generator (ENV-410C, Med Associates, Inc., St. Albans, VT). The third active lever press produced the delivery of 0.1 ml of 15% ethanol paired with a 5 s illumination of the cue light above the lever. If within a minute, animals did not complete an FR3 the green light turned off and the sequence was reinitiated. The resistance score was calculated as the average reward number of the first 10 min of the last three baseline sessions vs. the 10 min of the average reward number of the three punished schedule sessions [1].

AUD criteria distribution and addiction score

A rat was considered positive for an addiction-like criterion when its score for this behavior was in the 34% highest percentile of the distribution. The selection of this threshold was based on our previous work [1] and considering that a change of the selection threshold from 25% to 40% has minimal impact on the allocation of individual rats into groups [5]. We obtained four groups of rats depending on the number of positive criteria met (0crit, 1crit, 2crit and 3crit). The 0 and 3 criteria rats were defined as AUD-resilient and AUD-vulnerable rats respectively. The addiction score is calculated as the algebraic sum of the normalized score (z-score) of each criterion for each subject [6].

Ex Vivo Electrophysiology

Ex vivo whole-cell voltage clamp and current clamp recordings along with field potential recordings were conducted in 250 µm thick coronal brain slices containing layer 2/3 of the prelimbic cortex (PL). Brain slices containing the nucleus accumbens core (NacC) and the basolateral amygdala (BLA) were used as controls to evaluate the effects of LY-354740 in downstream regions. The experimental setup was carried out in a blinded fashion with both alcohol self-administering rats and water control groups running in parallel. One water control rat was excluded from the study due to displaying an abnormal stress response during handling.

Brain slice preparation

To obtain brain slices, rats were deeply anesthetized with isoflurane and decapitated. Brains were rapidly removed and transferred into a constantly oxygenated (95% O2, 5% CO2) modified artificial cerebrospinal fluid solution (aCSF) containing (in mM): 220 sucrose, 2 KCl, 0.2 CaCl2, 6 MgCl2, 26 NaHCO3, 1.3 NaH2PO4 and 10 D-glucose. Coronal brain slices (250 µm) containing the nAcS were obtained using a Leica VT 1200S Vibratome (Leica Microsystems AB, Bromma, Sweden), and submerged in a continuously oxygenated standard aCSF containing (in mM): 124 NaCl, 4.5 KCl, 2 CaCl2, 1 MgCl2, 26 NaHCO3, 1.2 NaH2PO4 and 10 D-glucose. After an incubation for 30 min in 33°C, slices were allowed to rest for additionally 30 min before electrophysiological recordings were performed. Slices were maintained at room temperature for the rest of the day [7].

Whole-cell recordings

A Nikon Eclipse FN-1 microscope equipped with a 10x/0.30 objective identified the PL cortex layer 2/3, and a 40×/0.80 water-immersion objective was used to identify glutamatergic pyramidal neurons for whole-cell recordings. Prelimbic pyramidal neurons were identified based on their typical firing pattern and they triangle-shaped soma. The morphology was confirmed by immunohistochemical identification of neurons filled with biocytin following electrophysiological recording. Briefly slices were post-fixed in 4% PFA in 1× PBS overnight at 4°C. Slices were then incubated in a solution of strpatavidin-594 (1:3000, Invitrogen) for 2 h in 1× PBS. Following 4 × 10 min PBS washes, slices were mounted onto Superfrost Plus glass slides (Epredia) and coverslipped with DAPI-Fluoromount-G aqueous mounting medium (Invitrogen). Cells showing atypical electrophysiological properties (e.g., different firing patterns, outliers in terms of basic membrane properties, i.e., Cm, Rm, and resting membrane potential) were excluded.

Recording pipettes were prepared from borosilicate glass using a micropipette puller (Sutter Instruments, Novato, CA) with a resistance ranging from 2.5 to 5.5 MΩ. Pipettes were filled with an internal solution containing (in mM): 135 K-Glu, 20 KCl, 2 MgCl2, 0.1 EGTA, 10 Hepes, 2 Mg-ATP and 0.3 Na-GTP, pH adjusted to 7.3 with KOH, and osmolarity to 295 mOsm with sucrose. Whole-cell recordings were conducted under constant flow (2 mL/min) of standard aCSF at the temperature of 33°C–34°C [8]. To record spontaneous excitatory postsynaptic currents (sEPSCs) in voltage clamp mode, neurons were clamped at-65 mV using a MultiClamp 700B amplifier (Molecular Devices, Axon CNS, San Jose, CA), digitized at 10 kHz and filtered at 2 kHz using Clampex (Molecular devices). In a subpopulation of these neurons, after the voltage clamp recording, a current clamp protocol was applied. Current was injected with a duration of 1,000 ms and an increasing intensity (in intervals of 20 pA) from −80 to 200 pA in order to hyperpolarize and depolarize the neuronal membrane. The input resistance was calculated from the average of responses to the hyperpolarizing current injections (from -80 pA to -20 pA).

Field potential recordings

In field potential recordings population spikes (PS) were evoked with a stimulation frequency of 0.05 Hz in the prelimbic cortex, nucleus accumbens and the basolateral amygdala [9]. Stimulation electrodes (type TM33B, World Precision Instruments, Sarasota, FL) were positioned locally, 0.2–0.3 mm from the recording electrode (borosilicate glass, 2.5 to 4.5 MΩ, World Precision Instruments), and the amplitude of PSs were measured. To assess changes in mGluR2/3 signaling, slices from PL, NacC and BLA were perfused with the mGluR2/3 agonist LY-354740 hydrate for (100nM). To monitor changes in inhibitory tone, slices from PL were treated with bicuculline (20 µM). When monitoring the responsiveness to LY-354740 and bicuculline, PS amplitude was set to half max response and a stable baseline was recorded for ten minutes before drugs were administered via bath perfusion. To assess changes in release probability after drug treatment, responses were evoked with a paired pulse stimulation protocol (0.1 Hz, 50-ms interpulse interval), and the paired pulse ratio (PPR) was calculated

by dividing the second pulse (PS2) with the first pulse (PS1). Signals were amplified by a custom-made amplifier, filtered at 3 kHz, digitized at 8 kHz (12-bit Analog-Digital converter with a maximum range of 10 V) and transferred to a PC for analysis.

Statistical analysis

Data are expressed as mean ± standard error with statistically significant difference set at P< .05. Prior to measures of analysis of variance (ANOVA) we examined for significant violations for assumptions of homogeneity of variance by using Levene's test. Mauchly's test of sphericity was used to test if assumption of sphericity had been violated when using repeated measures (RM)-ANOVA. For the behavioral data, lever responding during prolonged SA training was analyzed by RM-ANOVA with "group" as between-subject factor and "time" as within-subject factor. The discrimination between active and inactive lever was analyzed using RM-ANOVA with "time "as within-subject factor and "lever" as between-subject factor. One-way ANOVA was used to detect group effects in persistence of response, motivation, resistance to punishment and addiction score. A generalized linear mixed model (GLMM) analysis was used to evaluate timeout responding lever presses normalized by the number of rewards.

Significant main effects and interactions were further analyzed by a pair wise comparison of means using the Newman Keuls (NK) post hoc test.

For voltage-clamp electrophysiological measures of frequency, amplitude, rise and decay time were analyzed by one-way ANOVA. For current-clamp recordings one way-ANOVA was used to analyze membrane potential, threshold and rheobase while relative change in membrane potential evoked by current injection and AP frequency were analyzed with RM-ANOVA with "stimulation intensity "as within-subject factor and "criteria group" as between-subject factor. One way-ANOVA was used to detect group effects in passive membrane properties. If appropriate NK post hoc test was used. For field potential recordings a RM-ANOVA was used for comparisons of PS-amplitude after LY-354740, bicuculline bath perfusion, PPR and input/output function with "time" as within-subject factor and "criteria group" as between-subject factor.

All correlations present in the study were performed with Pearson's correlation analysis. Principal Component Analysis (PCA) was performed in Python with scipy v 1.7.3 and sklearn v 1.0.2. Data were first normalised with sklearn.preprocessing.StandardScaler, then PCA was performed with sklearn.decomposition.PCA.

Data were analyzed using STATISTICA, stat soft 13.0 (RRID:SCR_014213), Clampfit 10.2 (Molecular devices, Axon CNS, CA, United States), Minianalysis 6.0 (Synaptosoft), Microsoft Excel and GraphPad Prism 9 (GraphPad Software, San Diego, CA).

SUPPLEMENTARY FIGURES AND TABLES

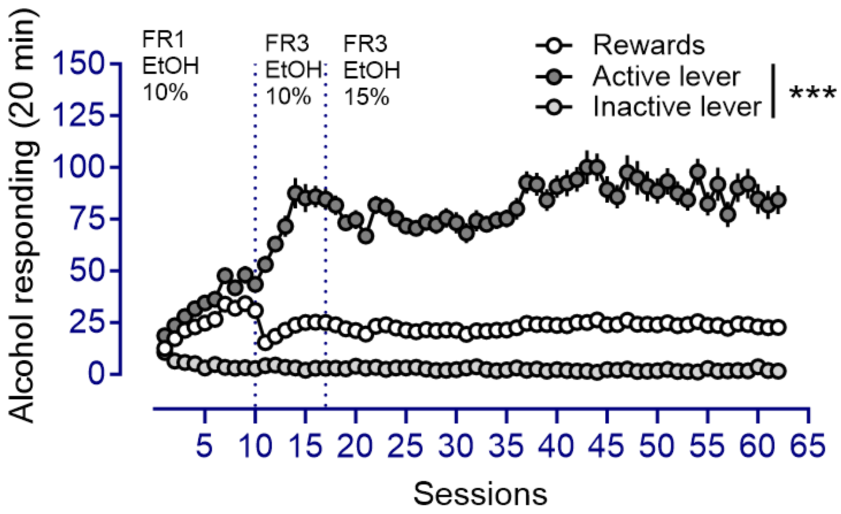

Figure S1: Rats (n=38) acquired and maintained stable alcohol self-administration levels under a FR-1 (day 1–9) and fixed ratio-3 (FR-3; day 9–62) schedule of reinforcement (***p<0.001 active lever vs inactive lever).

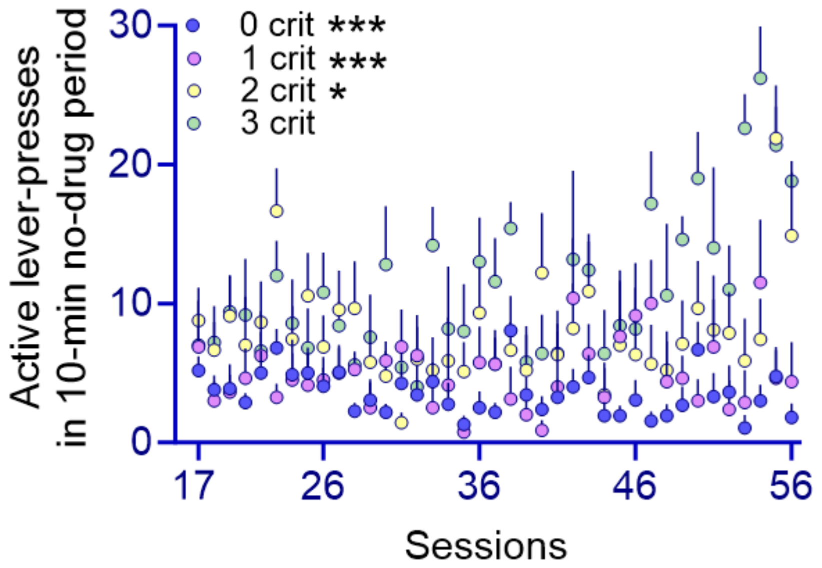

Figure S2: In persistence in responding in the 10-min no-drug period there was a significant effect of group ($F_{(1,3)}$=14.54; p<0.001), session ($F_{(3,34)}$=3.63; p<0.001) and group x session interaction ($F_{(3,34)}$=1.94; p<0.001). Post hoc analysis showed a significant higher active lever responding in 3crit rats as compared to 0crit (***p<0.001), 1crit (***p<0.001) and 2crit rats (*p<0.05).

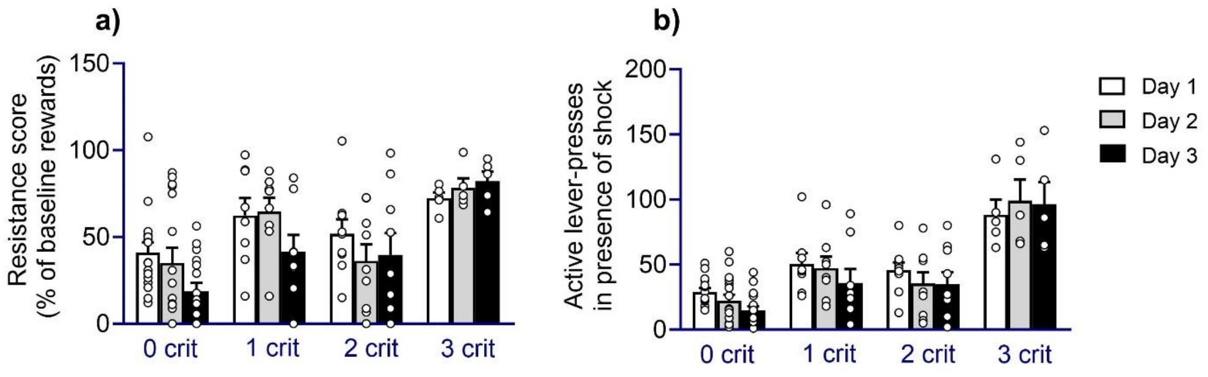

Figure S3: a) Resistance score values presented for the three-consecutive resistance to punishment sessions and divided by criteria group. Repeated measure ANOVA showed a significant effect of group ($F_{(3,34)}$=6.92, p<0.001), but not time ($F_{(2,68)}$=2,9 p=0.06), and group x time interaction ($F_{(2,68)}$=1.58, p=0.17). Post hoc Newman Keuls showed that 3crit rats had significantly higher active responding as compared to 0crit (p<0.001), 1crit (p<0.05) and 2crit rats (p<0.01). **b)** When considering the active lever presses in presence of shock for the three consecutive days there was a significant effect of group ($F_{(3,34)}$=16.26, p<0.001), time ($F_{(2,68)}$=3.67, p<0.05), but not group x time interaction ($F_{(2,68)}$=1.60, p=0.16). Active lever presses were significantly higher in the 3crit group as compared to 0crit, 1crit and 2crit rats (p<0.001).

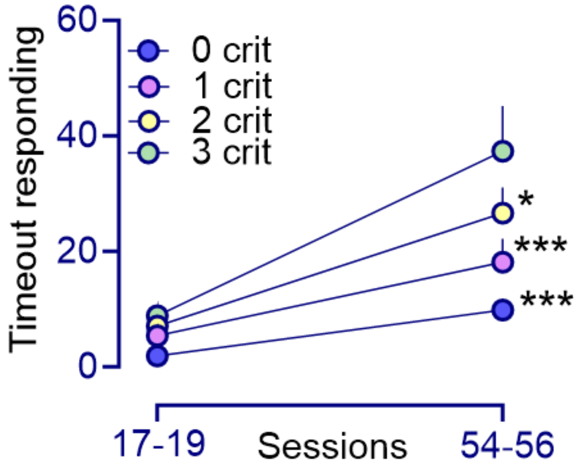

Figure S4. Timeout responding over time differed among groups ($F_{(3,34)}$= 9.89, p<0.001). Interestingly, while all groups started from similar levels in time-out responding in early operant training (0crit vs 1-2-3crit, p=ns; 1crit vs 2-3crit, p=ns and 2crit vs 3crit, p=ns), 3crit rats displayed significantly higher time-out responses compared to 0crit (p<0.001), 1crit (p<0.001) and 2crit (p<0.05) rats following extended training.

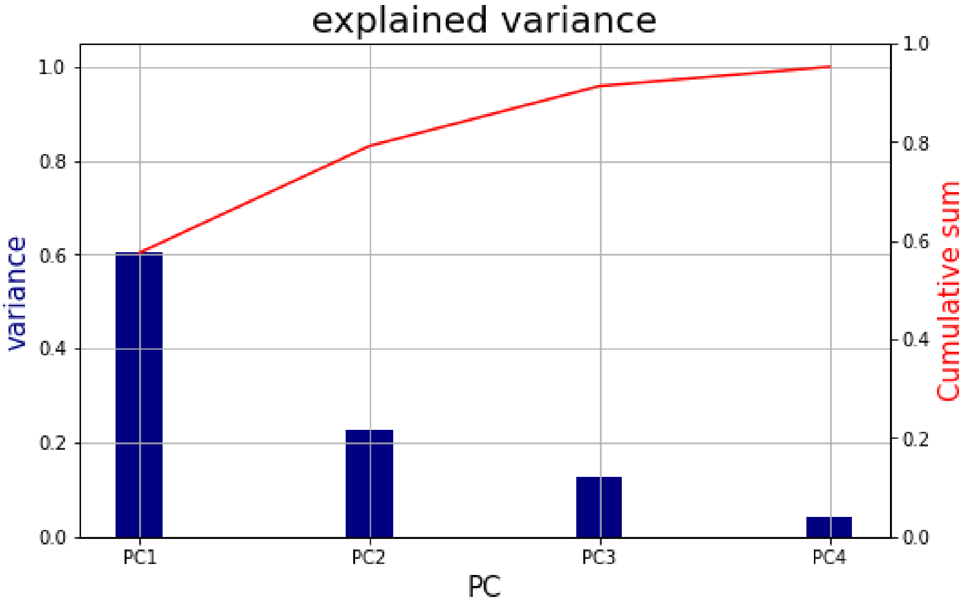

Figure S5. Principal component analysis: the blue bars show the percentage variance explained by each principal component (PC1, PC2, PC3 and PC4). The red line shows the cumulative sum.

Electrophysiological properties		Group Mean ± SEM					Statistical analysis
		Ctrl	0crit	1crit	2crit	3crit	Group effect
Voltage clamp	Frequency (Hz)	1.51 ±0.18	1.32 ±0.11	1.37 ±0.19	1.02 ±0.13	0.71± 0.09	$F_{(4,94)}$=4.39, p<0.01
	Amplitude (pA)	7.52 ±0.4	8.11 ±0.49	7.3 ±0.45	6.8± 0.26	8.66 ±1.07	$F_{(4,94)}$=1.78, p=0.14
	Rise Time (ms)	1.42 ±0.08	1.53 ±0.08	1.68 ±0.13	1.58 ±0.08	1.62 ±0.12	$F_{(4,94)}$=0.92, p=0.45
	Decay Time (ms)	5.87 ±0.35	6.23 ±0.28	6.38 ±0.45	6.08 ±0.25	7.41 ±0.71	$F_{(4,94)}$=1.94, p=0.11
Current clamp	Membrane potential (mv)	-63.45 ±1.67	-61.8 3±1.37	-67.56 ±1.95	-68.20 ±1.15	-60.34 ±2.34	$F_{(4,64)}$=4.33, p<0.01
	Threshold (mV)	-40.67 ±0.85	-40.01 ±1.33	-41.81 ±2.03	-42.31 ±1.61	-39.11 ±2.46	$F_{(4,64)}$=0.63, p=0.65
	Rheobase (pA)	113.3 ±17.96	102.5 ±14.82	125 ±18.93	129.4 ±17.67	72±20.3	$F_{(4,63)}$=1.99, p=0.10
Passive membrane	Input resistance (MΩ)	141.6 ±16.19	145.5 ±21.03	108.0 ±7.69	121.1 ±13.29	233.6 ±50.48	$F_{(4,64)}$=3.63, p≤0.01
	Capacitance (pF)	96.84 ±5.9	86.13 ±5.29	92.01 ±5.96	93.12 ±5.01	73.33 ±8.7	$F_{(4,94)}$=1.49, p=0.21

Table S1. Electrophysiological properties of layer 2/3 PL pyramidal neurons in control and 0-3crit rats.

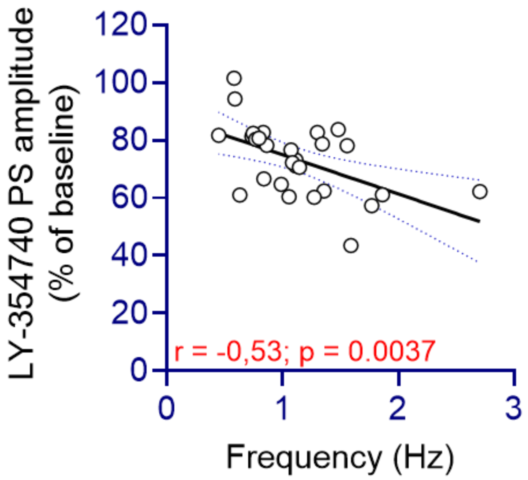

Figure S6. Significant correlation observed between the depression in PS amplitude induced by the mGluR2/3 agonist and the sEPSC frequency in voltage-clamp recordings in the recorded rats.

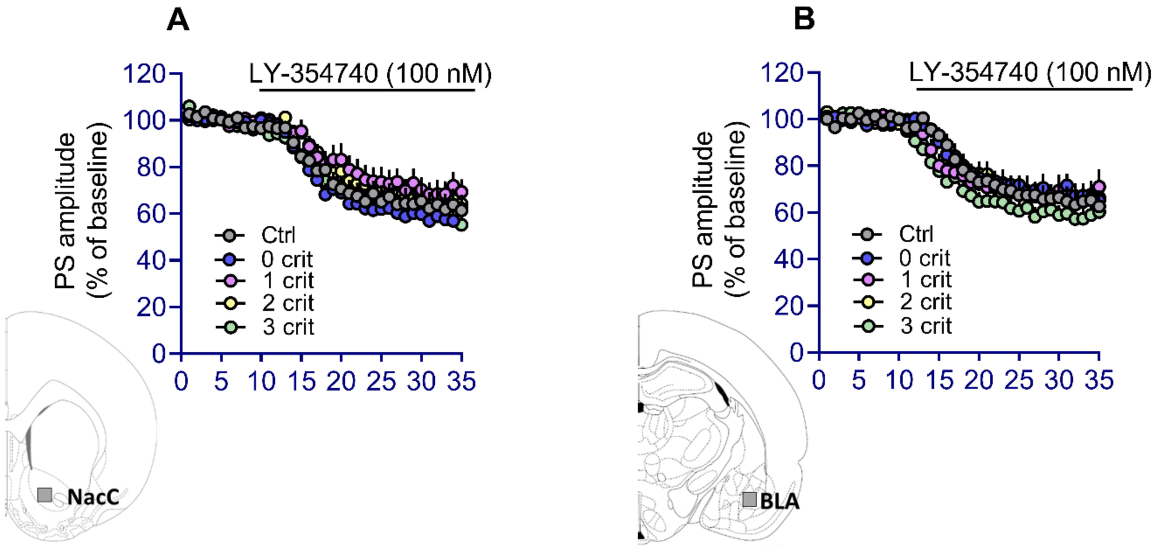

Figure S7. PS amplitude of evoked field potentials in the **(A)** NacC and **(B)** BLA during baseline (10 min) and bath application of the mGluR 2/3 agonist LY-354740 (25 min) with respective location site of recording.

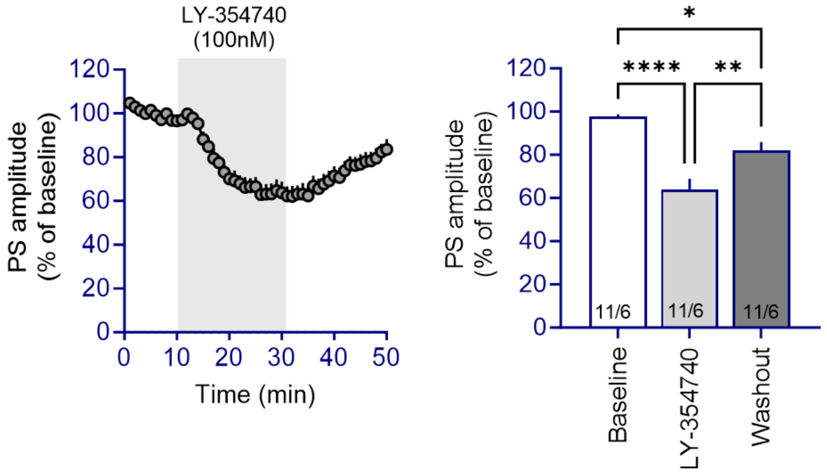

Figure S8. The decrease in PS amplitude following LY-354740 (100nM) is partially reversible upon washout (left figure: LY-354740 vs washout, p<0.01) (n = x/y; x: number of recordings/y: number of rats recorded).

SUPPLEMENTARY EXPERIMENTS

PL synaptic activity after footshock punishment sessions

Rationale

Using the multi-symptomatic 0/3 criteria model we identified sub-populations of rats exhibiting varying degrees of vulnerability and resilience in relation to alcohol addiction-like behaviors. Our electrophysiological findings revealed that both sEPSC frequency of glutamatergic prelimbic pyramidal neurons and the mGluR2/3 agonist-mediated synaptic depression of evoked potentials significantly correlated with the rats´ addiction score and as well with their resistance to punishment. It´ is worth noting that resistance to punishment is the last criterion we measure before the electrophysiological assessments. To mitigate potential bias from the footshock sessions themselves when examining neurophysiological adaptations, we examined the activity within the prelimbic cortex of rats subjected to three footshock punishment sessions. These rats were sacrificed for *ex vivo* electrophysiology approximately 4-5 days after the last footshock session, aligning with the alcohol rebaseline period used in our previous experiment. We performed both voltage-clamp recordings in pyramidal prelimbic neurons and evoked field potential recordings, with the PL slices bath-perfused with the mGluR2/3 agonist LY-354740.

Subjects

A new cohort (n=12) of Wistar rats (Charles River, Germany), weighing 230-250g at the start of the experiments, were used in this study. Rats were housed in pairs under a 12-hour reversed light/dark cycle (lights off at 8:00 AM). Food and water were provided ad libitum. Experiments were conducted during the dark phase of the cycle and all efforts were made to minimize rats' suffering and distress. At arrival, rats were left undisturbed to acclimatize at the animal facility for one week. The entire following week rats were handled twice a day prior to any behavioral procedure. Procedures were conducted in accordance with the National Committee for Animal Research in Sweden and approved by the Local Ethics Committee for Animal Care and Use at Gothenburg University (Dnr 5.8.18-13023/2019).

Drugs

The mGluR2/3 agonist LY-354740 hydrate (Tocris, Bristol, UK) was dissolved in Milli-Q to 5 mM and further diluted in artificial CSF (aCSF) to final concentration (100 nM).

Behavioral procedure

After a week of handling, rats were acclimated fort 10 min to the operant self-administration boxes for three consecutive days, with both levers retracted and no stimulus lights, meaning that rats were not engaged to perform any operant behavior. On the test day, they were randomly assigned to either the control (n=6) or foot-shocked (n=6) group. The test lasted 10 minutes, during which the foot-shocked group received passive foot-shocks matching the intensity (0.25 mA) and quantity of foot-shocks of rats that had previously shown resistance to punishment, while the control group served as a context, non-punished control. The test was repeated for three consecutive days mimicking the duration of punishment sessions presented in the main study.

Ex vivo electrophysiology

Please refer to material and methods described above.

Results: PL synaptic activity is not affected after footshock punishment sessions

Recordings performed in voltage clamp mode showed that the frequency (Student's *t*-test: $t_{(20)} = 0.14$, $p = 0.88$) and the amplitude ($t_{(20)} = 0.20$, $p = 0.83$) of spontaneous excitatory postsynaptic currents (sEPSCs) were not significantly affected by three consecutive foot-shock sessions. Furthermore, in the field potential recordings, there were no significant differences observed in synaptic depression of evoked potentials following pharmacological activation of mGluR2/3 between the control group and the group of rats that received yoked foot shocks. (group: $F_{(1, 26)} = 2.87$; $p=0.1$; time: $F_{(20, 500)} = 64.27$; $p<0.001$; time x group: $F_{(20,500)} = 0.85$; $p=0.65$). These results reinforce the notion that the glutamatergic adaptations observed in the AUD-vulnerable phenotype are a consequence of the emergence of addictive-like behaviors following prolonged alcohol consumption.

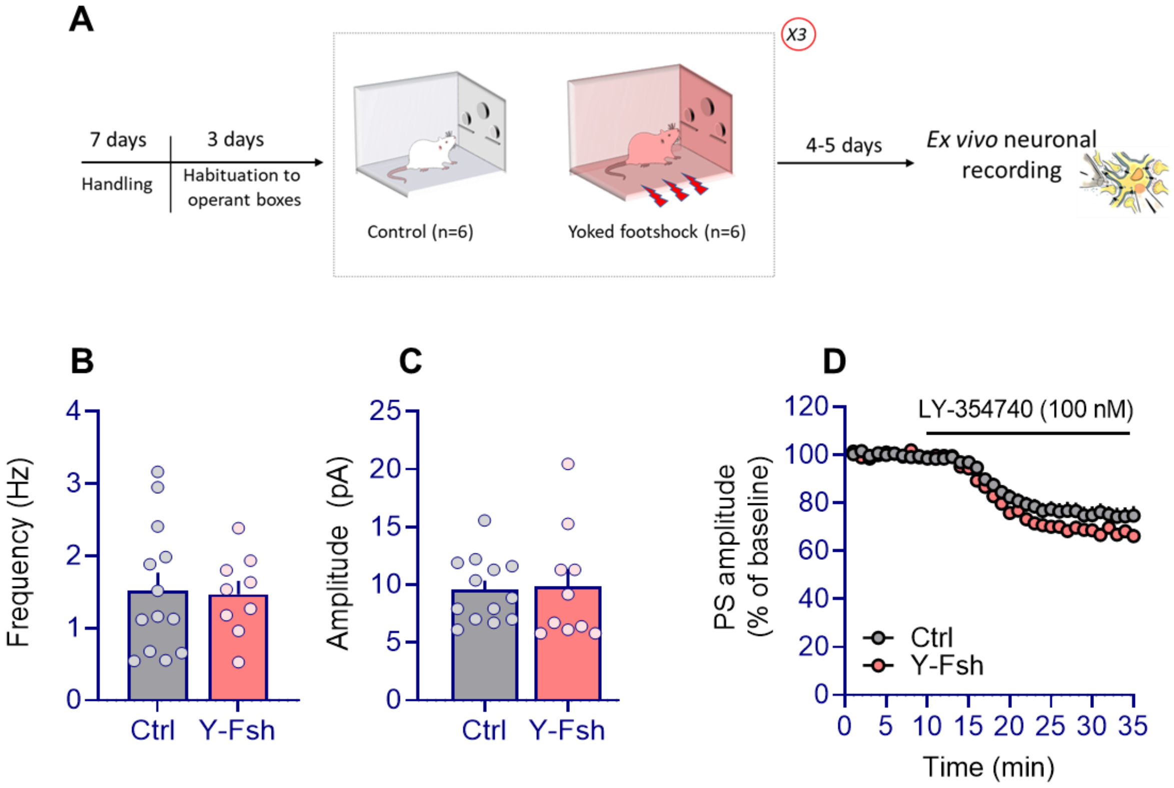

Figure S9. A) Timeline of the experimental procedures that include behavioral and neurophysiological assessments. **(B)** Voltage-clamp recordings of frequency (Hz) and **(C)** amplitude (pA) of spontaneous excitatory postsynaptic currents. **(D)** PS amplitude of synaptic depression of evoked potentials (Ps amplitude) by the mGlur 2/3 agonist LY-354740.

REFERENCES

1 Domi A, Stopponi S, Domi E, Ciccocioppo R, Cannella N. Sub-dimensions of Alcohol Use Disorder in Alcohol Preferring and Non-preferring Rats, a Comparative Study. Front Behav Neurosci. 2019;13:3.

2 Giuliano C, Peña-Oliver Y, Goodlett CR, Cardinal RN, Robbins TW, Bullmore ET, et al. Evidence for a Long-Lasting Compulsive Alcohol Seeking Phenotype in Rats. Neuropsychopharmacology. 2018;43(4):728-38.

3 Giuliano C, Belin D, Everitt BJ. Compulsive Alcohol Seeking Results from a Failure to Disengage Dorsolateral Striatal Control over Behavior. J Neurosci. 2019;39(9):1744-54.

4 García-Blanco A, Ramírez-López Á, Navarrete F, García-Gutiérrez MS, Manzanares J, Martín-García E, et al. Role of CB2 cannabinoid receptor in the development of food addiction in male mice. Neurobiol Dis. 2023;179:106034.

5 Deroche-Gamonet V, Piazza PV. Psychobiology of cocaine addiction: Contribution of a multi-symptomatic animal model of loss of control. Neuropharmacology. 2014;76 Pt B:437-49.
6 Belin D, Balado E, Piazza PV, Deroche-Gamonet V. Pattern of intake and drug craving predict the development of cocaine addiction-like behavior in rats. Biol Psychiatry. 2009;65(10):863-8.
7 Licheri V, Eckernäs D, Bergquist F, Ericson M, Adermark L. Nicotine-induced neuroplasticity in striatum is subregion-specific and reversed by motor training on the rotarod. Addict Biol. 2020;25(3):e12757.
8 Domi A, Lucente E, Cadeddu D, Adermark L. Nicotine but not saline self-administering or yoked control conditions produces sustained neuroadaptations in the accumbens shell. Front Mol Neurosci. 2023;16:1105388.
9 Domi A, Domi E, Lagstrom O, Gobbo F, Jerlhag E, Adermark L. Abstinence-Induced Nicotine Seeking Relays on a Persistent Hypoglutamatergic State within the Amygdalo-Striatal Neurocircuitry. eneuro. 2023;10(2):ENEURO.0468-22.2023.
